# Correlation between histopathological features and recurrence score according to menopausal status in HR+/HER2– breast cancer patients: a retrospective study

**DOI:** 10.37349/etat.2025.1002331

**Published:** 2025-07-18

**Authors:** Federica Martorana, Sabrina Nucera, Gianmarco Motta, Maria Vita Sanò, Carlo Carnaghi, Marialuisa Puglisi, Claudia Gelsomino, Giuseppe Corsaro, Chiara Conti, Lucia Motta, Giuliana Pavone, Stefano Marletta, Giada Maria Vecchio, Gaetano Magro, Giuseppe Catanuto, Gaetano Castiglione, Francesco Caruso, Antonio Rizzo, Michele Caruso, Paolo Vigneri

**Affiliations:** Cancer Research Program IMIM (Hospital del Mar Medical Research Institute), Spain; ^1^Department of Clinical and Experimental Medicine, University of Catania, 95123 Catania, Italy; ^2^Humanitas Istituto Clinico Catanese, Misterbianco, 95045 Catania, Italy; ^3^School of Specialization in Medical Oncology, Department of Human Pathology “G. Barresi”, University of Messina, 98125 Messina, Italy; ^4^Department of Medical and Surgical Sciences and Advanced Technologies, F. Ingrassia, University of Catania, 95123 Catania, Italy

**Keywords:** Oncotype DX, recurrence score, breast cancer, hormone-receptor positive

## Abstract

**Aim:**

Clinico-pathological features have traditionally guided prognosis and adjuvant therapy for breast cancer (BC) patients. In the past decade, genomic tests such as Oncotype DX entered clinical practice to refine risk stratification and predict chemotherapy benefit for hormone-receptor positive (HR+)/human epidermal growth factor-receptor 2 negative (HER2–) BC patients after surgery. This is a retrospective analysis to investigate the correlation between histopathological parameters and recurrence score (RS), accounting for menopausal status.

**Methods:**

Data on HR+/HER2– early BC patients who underwent Oncotype DX were collected using an institutional database. Clinico-pathological characteristics were retrieved. Linear regression was used with RS as a continuous outcome, while logistic regression was performed for pre- and post-menopausal patients, dichotomizing RS at thresholds of 16 and 25, respectively.

**Results:**

A total of 180 women were included (35% pre-menopausal, 65% post-menopausal). Median age was 57.5 years. Most patients had pT1, pN0, G2 BC, with median estrogen receptor (ER) expression of 95% and a median Ki67 of 25%. Median RS was 16 [interquartile range (IQR) 12–22] in the overall cohort, 15 in pre-menopausal, and 17 in post-menopausal women. In the entire cohort, RS significantly correlated with G3 (*P* = 0.01), Ki67% (*P* < 0.0001), ER% (*P* = 0.03), and progesterone receptor (PgR)% (*P* < 0.0001). In pre-menopausal patients, only Ki67% (*P* = 0.02), ER% (*P* = 0.01), and PgR% (*P* < 0.0001) showed significant correlations, while in post-menopausal patients, G3 (*P* = 0.03), Ki67% (*P* = 0.001), and PgR% (*P* < 0.0001) achieved statistical significance. Logistic regression analysis showed that in pre-menopausal patients, PgR% predicted RS > 16 [odds ratio (OR) 0.95, *P* = 0.001]. In post-menopausal women, Ki67% (OR 1.08, *P* = 0.031) and PgR% (OR 0.95, *P* < 0.0001) predicted RS > 25.

**Conclusions:**

In this patient cohort, classical clinico-pathological features showed varying correlations with RS, depending on menopausal status. These findings highlight the complexity of risk stratification, suggesting that further research is needed to better understand the factors influencing RS and its clinical utility.

## Introduction

In recent years, multigene tests such as Oncotype DX, MammaPrint, Breast Cancer Index, Prosigna (PAM50), and EndoPredict have transformed the management of hormone-receptor positive (HR+)/human epidermal growth factor-receptor 2 negative (HER2–) early breast cancer (BC). These tools are used to refine risk stratification and assess the potential benefit of chemotherapy for individual patients [1].

Oncotype DX is a genomic test that analyzes the expression of 21 genes using quantitative real-time polymerase chain reaction (qRT-PCR) from paraffin-embedded tumor tissue samples. The test result is expressed as a recurrence score (RS), which ranges from 0 to 100 [2]. The prognostic and predictive value of Oncotype DX has been extensively validated in both retrospective and prospective studies. The TAILORx trial showed that patients with HR+/HER2– node negative BC and a RS between 0 and 25 do not benefit from adjuvant chemotherapy [3, 4]. An exploratory analysis of the study demonstrated that subjects aged 50 or younger with a RS between 16 and 25 may derive an additional benefit from chemotherapy compared to endocrine therapy alone [5]. The role of Oncotype DX in HR+/HER2– BC patients with 1–3 positive locoregional lymph nodes (i.e. pN1) has been explored in the prospective RxPONDER trial. According to this study, adjuvant chemotherapy does not add a significant benefit to endocrine therapy alone in post-menopausal women with RS < 26, regardless of the tumor clinico-pathological parameters. However, in pre-menopausal pN1 patients with RS < 26, adjuvant chemotherapy seems to reduce the rate of distant recurrence, supporting its use in this population [6].

Despite the established role of Oncotype DX in the management of HR+/HER2– early BC patients, several factors, such as its cost and turn-around time, fostered interest in developing algorithms for RS prediction based on clinical parameters and tumor biomarkers. Many of these parameters, such as the immunohistochemistry (IHC) expression of estrogen receptor (ER) and progesterone receptor (PgR), HER2 status, tumor stage, lymph node involvement, and Ki67%, are already widely recognized for their prognostic and predictive roles [7]. However, data supporting RS prediction based on these factors are still limited and inconsistent, and none of these variables have been analyzed according to patient menopausal status. Still, previous evidence suggests that menopausal status can influence gene expression patterns in normal and cancer breast tissue, and that this could eventually impact genomic test results, including RS [8].

This study aims to explore the relationship between histopathological features and RS in patients with early HR+/HER2– BC, with a particular focus on the influence of menopausal status.

## Materials and methods

### Study population

We retrospectively analyzed a cohort of patients with a histologically confirmed diagnosis of early-stage HR+/HER2– BC who received an Oncotype DX test after curative surgery. All patients were followed at Humanitas Istituto Clinico Catanese (HICC) in Catania, Italy, from September 2021 (when the Oncotype DX test was made freely available from the Italian National Health System) to March 2024. Fully anonymized data were extracted from DataBreast, a prospectively maintained European Society of BC Specialists (EUSOMA)-approved institutional database for quality assurance [9, 10]. Patient’s informed consent was obtained before performing data entry.

Pathological examination, including IHC stainings, was performed on tumor tissues in the Pathology Unit of HICC. Tumor sections from formalin-fixed, paraffin-embedded specimens were sent to Genomic Health laboratories (Exact Sciences) to perform the Oncotype DX test.

Relevant information was extracted from the database, including age, menopausal status, baseline tumor pathological features, i.e. tumor size (T), axillary nodal status (N), histotype, HR status (ER, PgR), Ki67 values, HER2 status, lymphovascular invasion (LVI), perineural invasion (PNI), and Oncotype DX RS value. Only patients with complete clinico-pathological data were included in the analysis.

### Objectives

The primary aim of the study was to assess the association between traditional histopathological features and RS in patients with early-stage HR+/HER2– BC. Specifically, we examined how these variables influence RS values and contribute to risk stratification, with a focus on menopausal status.

### Statistical analysis

Continuous variables were reported as medians with interquartile range (IQR), while binary and categorical variables were expressed as percentages. For descriptive statistics, *t*-test and chi-square have been used for continuous and categorical variables, respectively.

Linear regression analysis was performed to assess the association between RS as a continuous variable and histopathological characteristics to identify potential predictive factors.

Logistic regression analysis was performed separately for pre- and post-menopausal patients, dichotomizing RS according to thresholds of 16 and 25, respectively, to evaluate the association between clinico-pathological characteristics and the likelihood of obtaining an RS above or below the clinically relevant thresholds.

All statistical analyses were performed using STATA software v18.0 (StataCorp., 2023, College Station, TX: StataCorp LLC).

## Results

### Demographics

We identified 187 patients with early-stage HR+/HER2– BC who underwent Oncotype DX testing at HICC between September 2021 and March 2024. Of these, 180 were included in the analysis.

Detailed clinico-pathological characteristics are reported in Table 1. Median age in the overall population was 57.5 years (IQR 49–67.5). Sixty-three patients (35%) were pre-menopausal, and 117 (65%) were post-menopausal. The majority of tumors (*n* = 132, 73.3%) had a non-special type (NST) histology, were moderately differentiated (i.e. G2) (*n* = 119, 66.1%), with a median tumor size of 18 mm (IQR 12.5–23), a pT1 stage (*n* = 111, 61.7%) and without nodal involvement (pN0, *n* = 111, 61.7%). Median ER expression was 95% (IQR 90–95), and median PgR expression was 80% (IQR 40–90). Approximately 40% of the population (*n* = 70, 38.9%) had a HER2 0 disease, the remaining 61.1% (*n* = 110) had a HER2 low (i.e. 1+ or HER2 2+ with a negative FISH test) tumor. The median Ki67 proliferation index was 25% (IQR 15–30). LVI was reported in 28.9% of patients (*n* = 52), while PNI was detected in 29.4% of cases (*n* = 53). Median RS in the overall population was 16 (IQR 12–22).

**Table 1:** Population characteristics

**Patient characteristics**	**Total population (*n* = 180, 100%)**	**Pre-menopausal (*n* = 63, 35.0%)**	**Post-menopausal (*n* = 117, 65.0%)**	***P*-value**
Age, years (median IQR)	57.5 (49–67.5)	47 (44–50)	64 (58–69)	**< 0.0001**
Tumor histology, *n* (%)				0.60
NST	132 (73.3)	49 (77.8)	83 (70.9)	
Lobular	32 (17.8)	9 (14.3)	23 (19.7)	
Other	16 (8.9)	5 (7.9)	11 (9.4)	
Tumor size, mm (median IQR)	18 (12.5–23)	18 (12–25)	18 (13–22)	0.57
pT, *n* (%)				0.50
pT1	111 (61.7)	41 (65.1)	70 (59.8)	
pT2	67 (37.2)	22 (34.9)	45 (38.5)	
pT3	2 (1.1)	-	2 (1.7)	
Number of positive nodes, *n* (%)				0.65
0	111 (61.7)	42 (66.7)	69 (59.0)	
1	55 (30.5)	18 (28.6)	37 (31.6)	
2	10 (5.6)	3 (4.7)	7 (6.0)	
3	2 (1.1)	-	2 (1.7)	
NA	2 (1.1)	-	2 (1.7)	
pN, *n* (%)				0.39
pN0	111 (61.7)	42 (66.7)	69 (59.0)	
pN1	67 (37.2)	21 (33.3)	46 (39.3)	
NA	2 (1.1)	-	2 (1.7)	
LVI, *n* (%)				0.47
Evident	52 (28.9)	20 (31.8)	32 (27.3)	
Not evident	125 (69.4)	41 (65.0)	84 (71.8)	
NA	3 (1.7)	2 (3.2)	1 (0.9)	
PNI, *n* (%)				0.87
Present	53 (29.4)	17 (27.0)	36 (30.7)	
Abstent	120 (66.7)	40 (63.5)	80 (68.4)	
NA	7 (3.9)	6 (9.5)	1 (0.9)	
Tumor grading, *n* (%)				0.21
1	12 (6.7)	3 (4.8)	9 (7.7)	
2	119 (66.1)	47 (74.6)	72 (61.5)	
3	49 (27.2)	13 (20.6)	36 (30.8)	
ER positivity, % (median IQR)	95 (90–95)	95 (90–95)	95 (90–95)	**0.0002**
PgR positivity, % (median IQR)	80 (40–90)	90 (55–90)	70 (30–90)	**0.0051**
HER2, *n* (%)				0.42
0	70 (38.9)	22 (34.9)	48 (41.0)	
1–2	110 (61.1)	41 (65.1)	69 (59.0)	
Ki67, % (median IQR)	25 (15–30)	20 (15–28)	25 (15–30)	0.29
RS (median IQR)	16 (12–22)	15 (11–18)	17 (12–22)	0.41

Bold indicates statistically significant *P*-values (≤ 0.05). IQR: interquartile range; NST: non-special type; NA: not available; LVI: lymphovascular invasion; PNI: perineural invasion; ER: estrogen receptor; PgR: progesterone receptor; HER2: human epidermal growth factor-receptor 2; RS: recurrence score

No significant differences were observed between the pre- and post-menopausal patients, except for age, which was younger in pre-menopausal women. ER expression had a superimposable mean and IQR in the two groups, indicating an uneven distribution at extreme values (range min–max: 60–98% in pre-menopausal individuals; 80–100% in post-menopausal women). PgR levels were higher in the pre-menopausal group.

**Figure 1:**
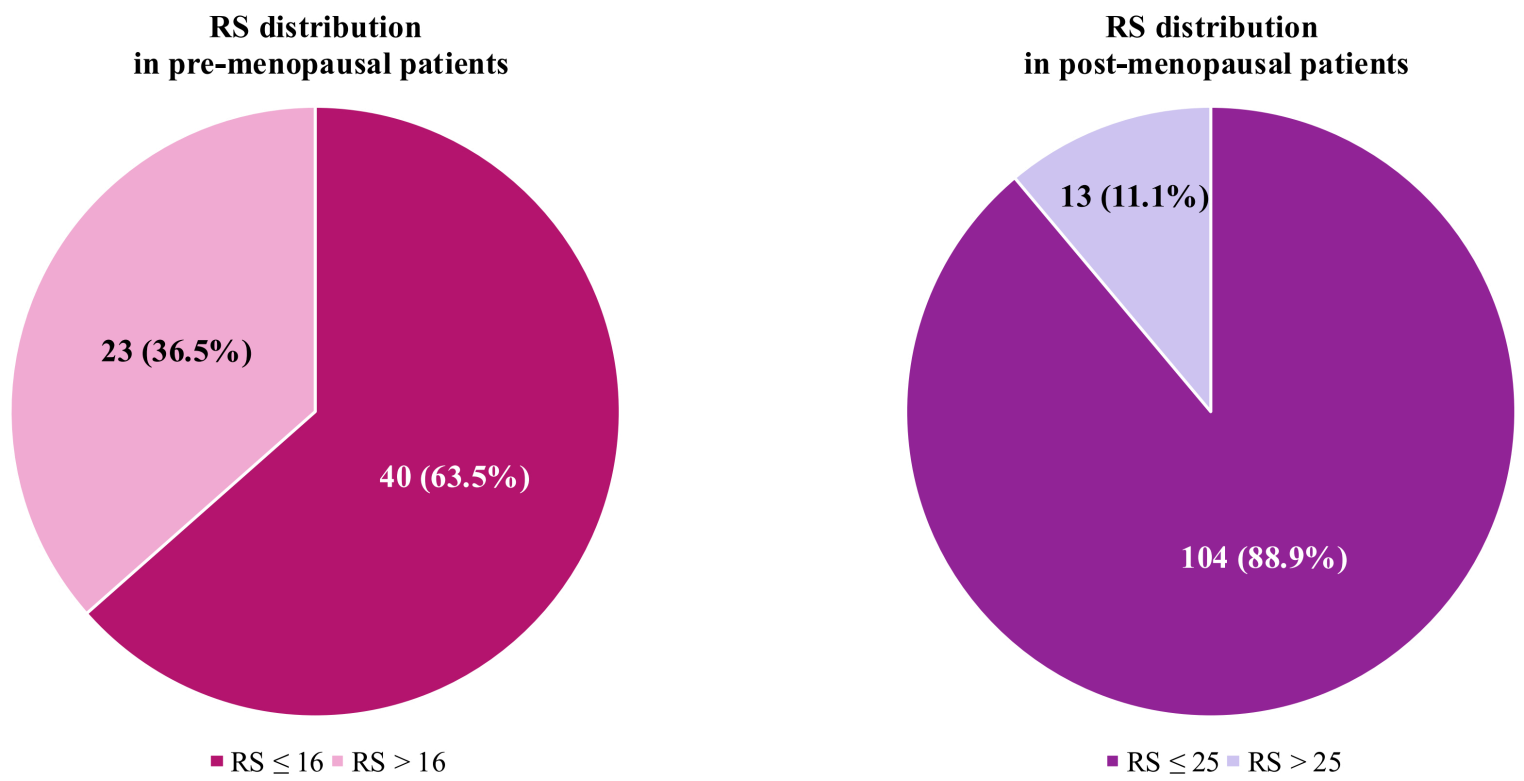
Recurrence score (RS) distribution according to menopausal status, using the standard threshold for high-risk definition

Median RS was slightly lower in pre-menopausal patients (median RS = 15, IQR 11–18) compared to post-menopausal subjects (median RS = 17, IQR 12–22), but this difference was not statistically significant. Among pre-menopausal women, 36.5% (*n* = 23) had an RS > 16, exceeding the threshold for high-risk definition. In the post-menopausal cohort, only 11.1% (*n* = 13) had a RS > 25, identifying them as high-risk (Figure 1).

### Pathological features and RS

We performed linear regression analysis on the entire cohort to correlate RS, considered as a continuous variable, with several pathological variables (Table 2).

**Table 2:** Linear regression analysis of recurrence score as a continuous variable and classical pathological features in the total population

**Variable**	**Coefficient**	**95% CI**	***P*-value**
Histology			
NST	-	-	-
Lobular	–1.10	–4.41 to 2.20	0.51
Other	–1.36	–5.79 to 3.08	0.55
LVI	0.10	–2.68 to 2.89	0.94
PNI	–0.69	–3.42 to 2.05	0.62
Grading			
1	-	-	-
2	3.10	–1.86 to 8.06	0.22
3	6.70	1.43 to 11.97	**0.01**
ER%	–0.29	–0.54 to –0.04	**0.03**
PgR%	–0.14	–0.18 to –0.11	**< 0.0001**
HER2			
0	-	-	-
1–2	1.08	–1.47 to 3.63	0.41
Ki67%	0.28	0.15 to 0.40	**< 0.0001**

Bold indicates statistically significant *P*-values (≤ 0.05). CI: confidence interval; NST: non-special type; LVI: lymphovascular invasion; PNI: perineural invasion; ER: estrogen receptor; PgR: progesterone receptor; HER2: human epidermal growth factor-receptor 2

In the overall cohort, G3 [confidence interval (CI) 95%: 1.43–11.97; *P* = 0.01] and Ki67% (CI 95%: 0.15–0.40; *P* < 0.0001) had a significantly positive correlation with RS, while ER (CI 95%: –0.54 to –0.04; *P* = 0.03) and PgR expression (CI 95%: –0.18 to –0.11; *P* < 0.0001) displayed a significantly inverse association with RS.

When stratifying the population by menopausal status, some differences emerged (Table 3). In pre-menopausal women, RS remained significantly correlated with Ki67 (*P* = 0.02), ER (CI 95%: –0.71 to –0.10; *P* = 0.01), and PgR expression (CI 95%: –0.24 to –0.12; *P* < 0.0001). In the post-menopausal group, statistical significance persisted for G3 (CI 95%: 0.55–13.22; *P* = 0.03), Ki67 (CI 95%: 0.13–0.44; *P* = 0.001), and PgR expression (CI 95%: –0.18 to –0.09; *P* < 0.0001).

**Table 3:** Linear regression analysis of the recurrence score as a continuous variable and classical pathological features in pre- and post-menopausal patients

**Pre-menopausal patients**				**Post-menopausal patients**			
**Variable**	**Coefficient**	**95% CI**	***P*-value**	**Variable**	**Coefficient**	**95% CI**	***P*-value**
Histology				Histology			
NST	-	-	-	NST	-	-	-
Lobular	0.26	–5.56 to 6.08	0.93	Lobular	–1.78	–5.87 to 2.31	0.39
Other	–4.61	–12.14 to 2.93	0.23	Other	0.03	–5.53 to 5.59	0.99
LVI	1.52	–2.94 to 5.97	0.50	LVI	–0.62	–4.23 to 2.99	0.73
PNI	–1.09	–5.52 to 3.33	0.62	PNI	–0.52	–4.01 to 2.97	0.77
Grading				Grading			
1	-	-	-	1	-	-	-
2	1.11	–78.25 to 10.46	0.81	2	4.06	–1.95 to 10.10	0.184
3	6.08	–3.98 to 16.14	0.23	3	6.89	0.55 to 13.22	**0.03**
ER%	–0.41	–0.71 to 0.10	**0.01**	ER%	–0.24	–0.69 to 0.21	0.30
PgR%	–0.18	–0.24 to –0.12	**< 0.0001**	PgR%	–0.14	–0.18 to –0.09	**< 0.0001**
HER2				HER2			
0	-	-	-	0	-	-	-
1–2	–1.66	–5.93 to 2.54	0.43	1–2	2.59	–0.63 to 5.82	0.11
Ki67%	0.26	0.05 to 0.47	**0.02**	Ki67%	0.28	0.13 to 0.44	**0.001**

Bold indicates statistically significant *P*-values (≤ 0.05). CI: confidence interval; NST: non-special type; LVI: lymphovascular invasion; PNI: perineural invasion; ER: estrogen receptor; PgR: progesterone receptor; HER2: human epidermal growth factor-receptor 2

Next, we analyzed RS as a categorical variable, applying the standard high-risk thresholds (> 16 for pre-menopausal and > 25 for post-menopausal patients) (Table 4). PgR expression showed a significantly inverse correlation in both groups [odds ratio (OR) 0.95; 95% CI: 0.93–0.98; *P* = 0.001 in pre-menopausal patients; OR 0.95; 95% CI: 0.93–0.98; *P* < 0.0001 in post-menopausal women]. However, in post-menopausal women, Ki67% also displayed a significant correlation with RS > 25 (OR 1.08; 95% CI: 1.01–1.16; *P* = 0.031).

**Table 4:** Logistic regression analysis of the recurrence score (RS) as a categorical variable using standard thresholds for high-risk definition according to menopausal status

**Pre-menopausal patients (RS threshold 16)**				**Post-menopausal patients (RS threshold 25)**			
**Variable**	**Odds ratio**	**95% CI**	***P*-value**	**Variable**	**Odds ratio**	**95% CI**	***P*-value**
Histology				Histology			
NST	-	-	-	NST	-	-	-
Lobular	1.51	0.37 to 6.36	0.58	Lobular	1.00	-	-
Other	1.25	0.19 to 8.25	0.81	Other	0.59	0.07 to 5.05	0.63
LVI	0.67	0.21 to 2.10	0.49	LVI	0.77	0.20 to 2.98	0.70
PNI	0.69	0.20 to 2.36	0.56	PNI	0.64	0.16 to 2.47	0.51
Grading				Grading			
1–2	-	-	-	1–2	-	-	-
3	1.66	0.48 to 5.74	0.42	3	1.47	0.45 to 4.86	0.53
ER%	0.93	0.85 to 1.02	0.11	ER%	1.02	0.86 to 1.21	0.81
PgR%	0.95	0.93 to 0.98	**0.001**	PgR%	0.95	0.93 to 0.98	**< 0.0001**
HER2				HER2			
0	-	-	-	0	-	-	-
1–2	0.75	0.26 to 2.18	0.60	1–2	2.54	0.66 to 9.78	0.18
Ki67%	1.06	0.10 to 1.12	0.061	Ki67%	1.08	1.01 to 1.16	**0.031**

Bold indicates statistically significant *P*-values (≤ 0.05). CI: confidence interval; NST: non-special type; LVI: lymphovascular invasion; PNI: perineural invasion; ER: estrogen receptor; PgR: progesterone receptor; HER2: human epidermal growth factor-receptor 2

**Figure 2:**
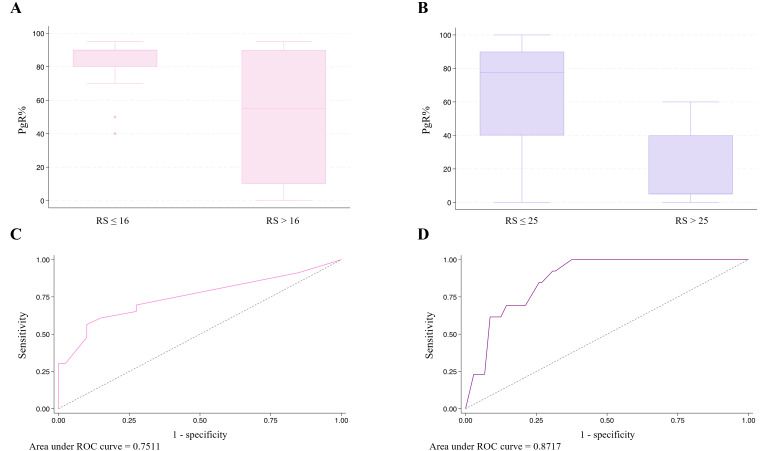
Progesterone receptor (PgR) distribution according to recurrence score (RS) category in pre-menopausal (A) and post-menopausal (B) patients, and the area under receiver operating characteristic (ROC) curve for RS prediction based on PgR levels in pre-menopausal (C) and post-menopausal (D) patients

Since PgR expression was the only variable consistently showing a significantly inverse correlation across all analyses (both linear and logistic regression), we assessed its predictive performance using the area under the receiver operating characteristic (ROC) curve. In pre-menopausal patients, the area under the curve (AUC) was 0.75, whereas in post-menopausal patients, it was 0.87, indicating different predictive performances between the two groups (Figure 2).

## Discussion

Genomic tests, such as Oncotype DX, are widely used in clinical practice to assess the risk of recurrence in patients with early-stage HR+/HER2– BC [11, 12]. These tests assist therapeutic choices in the adjuvant setting by identifying individuals who can safely forgo chemotherapy, thus avoiding unnecessary and potentially toxic treatments [13]. Results from pivotal trials, such as TAILORx and RxPONDER, suggest that menopausal status should be taken into account when interpreting Oncotype DX results, since it has an impact on chemotherapy efficacy in HR+/HER2– BC [8].

Over the years, different studies explored pathological features that, alone or in combination, could surrogate RS in clinical practice [14–18]. However, none of these studies incorporated a subgroup analysis according to patient menopausal status.

We retrospectively evaluated a cohort of HR+/HER2– early BC patients treated at a single institution who underwent Oncotype DX for risk definition and adjuvant treatment decision-making, looking for pathological features correlated to RS in the overall population and in the pre- and post-menopausal subgroups.

In our cohort, clinical and pathological characteristics were superimposable in the two populations, with the only exception of ER and PgR expression levels, which were higher in pre-menopausal women. RS was also evenly distributed between pre- and post-menopausal patients, with a median value of 16 in the overall cohort, 15 in the pre- and 17 in the post-menopausal population. These results are in line with those by Carr et al. [19], who conducted a retrospective analysis on 575 HR+/HER2– early BC patients, finding higher ER levels in pre-menopausal compared to post-menopausal patients, but comparable RS values in the two groups.

We then applied different RS thresholds to identify high-risk individuals, according to menopausal status (i.e. RS > 16 and > 25 for pre- and post-menopausal women, respectively), showing that a larger proportion of patients in the pre-menopausal group presented a high risk of recurrence (36.5%) compared to post-menopausal women (11.1%). This result suggests that, although the optimal interpretation of RS in pre-menopausal women remains highly debated, younger patients diagnosed with HR+/HER2– BC are more likely to benefit from adjuvant chemotherapy [20].

According to the results of the RxPONDER trial, node positive pre-menopausal patients derive an invasive disease-free survival and distant recurrence-free survival benefit regardless of RS [6]. Hence, American Society of Clinical Oncology (ASCO) guidelines suggest avoiding offering Oncotype DX to pre-menopausal women with 1–3 positive nodes to guide decisions on adjuvant therapy [21]. Still, in clinical practice, the test is widely used even in this subgroup of patients as suggested by real world evidence [22], and as shown by our study, in which 33.3% of pre-menopausal women had pN1 disease, mostly due to the presence of a single positive node. The ongoing OFSET trial (NCT05879926) will clarify whether endocrine therapy plus ovarian function suppression (OFS) will yield comparable results to chemotherapy followed by endocrine therapy plus OFS in pre-menopausal patients with pN1 HR+/HER2– early BC and an RS of 0–25.

In our study, among the considered pathological parameters, tumor grading, Ki67, ER, and PgR displayed a significant correlation with RS, with some differences according to menopausal status.

Having G3 BC was associated with higher continuous RS values in the overall population and in the post-menopausal subgroup, but not in pre-menopausal patients. Additionally, no correlation emerged between grading and RS when considering the latter as a dichotomous outcome. Tumor grade is a known independent marker of BC aggressiveness, and it also emerged as a significant predictor of RS in several retrospective series [14, 15, 23]. Grading is also incorporated, along with patient age and tumor size, in the RSClin score, a tool developed to refine, rather than surrogate, Oncotype DX results [24].

In our analysis, the Ki67 proliferation index showed a direct association with continuous RS values, regardless of menopausal status. This correlation persisted only in post-menopausal women when RS was considered as a binary outcome. Like grading, Ki67—assessed by IHC—has also been associated with BC prognosis [25]. However, evidence of a direct correlation with RS is less straightforward. In a study by Patel et al. [26], no association emerged between Ki67 and RS in a retrospective cohort of 525 women. Additional evidence suggests only a moderate positive correlation between Ki67 and RS [27, 28]. Of note, the *MKI67* gene, encoding for Ki67, is one of the proliferation-associated genes evaluated by Oncotype DX. However, a report by Selmani et al. [29] demonstrated a modest correlation between IHC assessment of Ki67 and Ki67 expression at the RNA level quantified with Oncotype DX. Other evidence suggests that Ki67 could be useful in surrogating RS, especially when combined with further pathological parameters [14, 30].

ER and PgR levels were negatively associated with RS in our study, with the latter displaying the strongest and most consistent results. The Oncotype DX assay quantifies both ER and PgR RNA, and several papers have investigated the concordance between protein and transcript expressions, finding an overall good agreement but a superior sensitivity for IHC [17, 31, 32].

In our cohort, using linear regression analysis, lower ER levels seem to predict higher RS values, especially in pre-menopausal patients. This correlation was lost in the post-menopausal subgroup after RS dichotomization. This result is in line with an exploratory analysis of the large phase III PlanB trial, where IHC ER expression had a weak negative correlation with RS [28]. Of note, in pre-menopausal women, ER expression at both mRNA and protein levels may fluctuate during different phases of the menstrual cycle, making their correlation with RS difficult to interpret [33–35]. However, clear evidence suggesting that the menstrual cycle phase can significantly influence RS is still lacking.

Among all the considered pathological features, PgR expression was the only parameter demonstrating a persistently significant negative correlation with RS, regardless of menopausal status, with better performance in the post-menopausal cohort according to a ROC analysis. PgR expression has a well-known prognostic role in BC, with lower levels predicting inferior outcomes. Several thresholds to discriminate between PgR-low and -high tumors have been proposed over time, with inconsistent results thus far [36, 37]. Stratifying our population according to PgR levels < 10 or > 10, we obtained a significant inverse correlation with RS (data not shown), even though the small number of patients in each subgroup did not allow us to draw definitive conclusions. In general, our results are in line with pre-existing literature demonstrating that PgR is a valid surrogate for RS, either alone or combined with other variables [15, 16, 30, 38].

Our study presents some limitations. Firstly, our cohort is not large enough to fully generalize the results to all patients with HR+/HER2– early BC. This is especially true for the pre-menopausal cohort, which comprised only 63 patients. Additionally, being a retrospective analysis, the available information is based on pre-existing clinical data, which could introduce selection bias. In this context, the definition of menopausal status, as self-reported by patients, could be heavily biased. This limitation has been extensively discussed for large randomized trials, such as TAILORx and RxPONDER [8]. Over time, more precise and robust methods to define menopausal status have been investigated, including measurement of the anti-Mullerian hormone, but their application in clinical practice is still limited [39]. IHC evaluation could also be biased in the context of a retrospective analysis, due to the subjectivity of the assessment. However, all the evaluations in our study have been performed at a single institution, minimizing the risk of inter-observer variability. Another significant shortcoming of our work is the lack of long-term follow-up, which prevents us from definitively assessing the predictive ability of Oncotype DX for recurrence.

Taking into account these inherent limitations, our findings suggest that risk stratification based on clinical-pathological factors, such as tumor grade, Ki67, ER, and PgR, could be useful in personalizing treatment according to patient menopausal status. The ability of these clinical parameters to predict the RS could support the choice of the test, guiding physicians to select patients who will benefit most from a molecular evaluation. Further prospective studies with longer follow-up and a larger cohort are needed to further validate these results and improve the accuracy of risk stratification for women with HR+/HER2– early BC. In this context, a significant effort should be made to generate results that may help to refine treatment and follow-up indications according to the menopausal status of these patients.

## Abbreviations

BC: breast cancer
CI: confidence interval
ER: estrogen receptor
HER2–: human epidermal growth factor-receptor 2 negative
HICC: Humanitas Istituto Clinico Catanese
HR+: hormone-receptor positive
IHC: immunohistochemistry
IQR: interquartile range
OR: odds ratio
PgR: progesterone receptor
RS: recurrence score

## Declarations

### Availability of data and materials

The raw data supporting the conclusions of this manuscript will be made available by the authors, without undue reservation, to any qualified researcher.

### Copyright

© The Author(s) 2025.
